# Dual-energy CT biomarkers for predicting the efficacy of TACE combined with lenvatinib and immune checkpoint inhibitors in unresectable HCC

**DOI:** 10.1186/s41747-025-00669-9

**Published:** 2026-01-19

**Authors:** Jingwen Zhang, Kai Zhang, Taoming Du, Cheng Yan, Yingxuan Wang, Mingzi Gao, Jing Han, Mingxin Zhang, Yujie Chen, Liqin Zhao

**Affiliations:** 1 Department of Radiology, Beijing Tiantan Hospital, Capital Medical University, Beijing, China; 2 Department of Radiology, Qilu Hospital of Shandong University, Jinan, China; 3 Department of Radiology, Chengdu Seventh People’s Hospital, Affiliated Cancer Hospital of Chengdu Medical College, Chengdu, China

**Keywords:** Carcinoma (hepatocellular), Chemoembolization (therapeutic), Immune checkpoint inhibitors, Lenvatinib, Tomography (x-ray computed)

## Abstract

**Objectives:**

To develop a nomogram based on low-dose one-stop dual-energy and perfusion computed tomography (LD-DE&PCT) for predicting the efficacy of transcatheter arterial chemoembolization (TACE) combined with lenvatinib and immune checkpoint inhibitors (TACE-LEN-ICIs) in unresectable hepatocellular carcinoma (uHCC) patients.

**Materials and methods:**

This prospective, multicenter study included uHCC patients who underwent LD-DE&PCT scanning. The relationships between quantitative LD-DE&PCT-derived parameters and the efficacy of TACE-LEN-ICIs were analyzed using logistic regression analysis. A nomogram incorporating the independent predictors was constructed, and its predictive performance was evaluated by the area under the receiver operating characteristic curve (AUROC).

**Results:**

A total of 125 lesions from 71 uHCC patients were enrolled, with 71 lesions (56.8%) classified as the objective response (ObR) group and 54 lesions (43.2%) as the non-response (NR) group. Univariate analysis revealed significant differences in tumor size, corona enhancement, tumor location, iodine concentration in the arterial phase (IC-AP), normalized iodine concentration in the arterial phase (NIC-AP), effective atomic number in the arterial phase (Z_eff_-AP), slope of spectral HU curve in the arterial phase (λ_HU_-AP), and permeability surface area product (PS) between ObR and NR groups. Among these, NIC-AP exhibited the highest predictive value (AUROC = 0.770; 95% confidence interval [CI]: 0.682‒0.858). Multivariate analysis identified tumor size, NIC-AP, and PS as independent predictors. The nomogram showed excellent performance (AUROC  = 0.913; 95% CI: 0.858–0.968). The total radiation dose was 19.02 ± 5.39 mSv.

**Conclusion:**

The LD-DE&PCT-based nomogram can accurately predict the response to TACE-LEN-ICIs in uHCC patients.

**Relevance statement:**

Low-dose one-stop dual-energy and perfusion CT provides a noninvasive method to predict response to TACE combined with lenvatinib and immune checkpoint inhibitors in unresectable HCC.

**Key Points:**

- Predicting response to TACE-LEN-ICIs in uHCC helps treatment decision-making.
- NIC-AP and PS from LD-DE&PCT, and tumor size were independent predictive biomarkers.
- NIC-AP was the best parameter for predicting response to TACE-LEN-ICIs in uHCC.

**Graphical Abstract:**

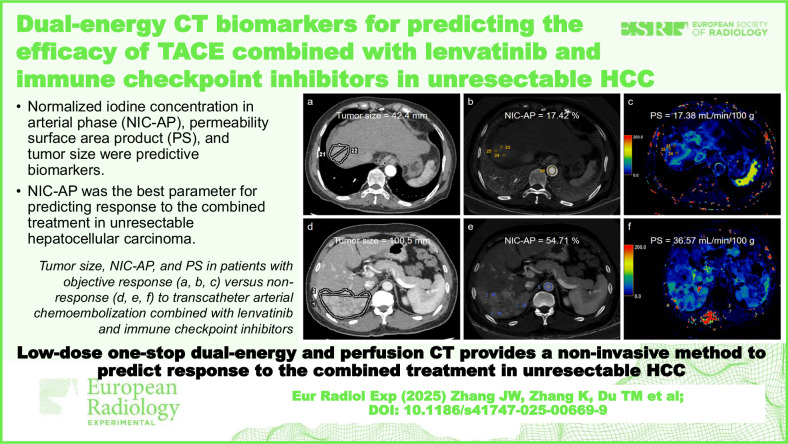

## Background

Established guidelines recommend transcatheter arterial chemoembolization (TACE) as the standard locoregional therapy for intermediate-stage hepatocellular carcinoma (HCC), while tyrosine kinase inhibitors (TKIs) and immune checkpoint inhibitors (ICIs) serve as the primary systemic treatments for advanced-stage HCC [1, 2]. Recent clinical trials have shown that the combination of TACE with TKIs and ICIs significantly improves prognosis compared with other treatments in unresectable HCC (uHCC) [3, 4]. Lenvatinib has replaced sorafenib as the first-line TKI due to its stronger synergistic effects when combined with TACE and its controllable adverse effects [5, 6]. However, the response to TACE combined with lenvatinib and ICIs (TACE-LEN-ICIs) varies substantially due to tumor heterogeneity and diverse histological subtypes, with objective response (ObR) rates ranging from 38.57% to 76.70% among uHCC patients [7–12]. Meanwhile, a positive treatment response is associated with longer overall and progression-free survival [13], highlighting the importance of accurately predicting the efficacy of TACE-LEN-ICIs. Moreover, this combined locoregional and systemic strategy may also increase the likelihood of treatment-related adverse effects, which lead to unfavorable outcomes in uHCC patients. Therefore, identifying patients who are most likely to benefit from this regimen is crucial for optimizing therapeutic strategies and improving outcomes across different efficacy groups.

Recently, limited studies have explored biomarkers for predicting the response to TACE-LEN-ICIs in uHCC patients, as this combination therapy remains an emerging treatment strategy. Most previous studies have focused on predicting the response to monotherapy or dual therapy in HCC. In the field of artificial intelligence, only a limited number of studies have developed radiomics-based models to predict the response to TACE-LEN-ICIs in uHCC patients [14, 15]. Although radiomics holds significant potential, its reliance on complex image processing and high-dimensional analysis hinders widespread clinical implementation. These limitations lead to inconsistent results and unsatisfactory diagnostic accuracy, emphasizing the necessity and urgency to develop a novel imaging biomarker.

Dual-energy computed tomography (DECT) utilizes different energy spectra to differentiate tissues based on their specific attenuation properties at different X-ray energies [16]. DECT can generate functional images such as material density images, virtual monochromatic images, spectral curves, and effective atomic number (Z_eff_) maps, which are not achievable with conventional CT. Previous studies have demonstrated the potential value of DECT in characterizing HCC and predicting microvascular invasion [16, 17]. Perfusion computed tomography (PCT) is another functional imaging technique that quantitatively assesses tumor vascularity and angiogenesis [18]. Recent research has shown that specific PCT-derived parameters can reflect hemodynamic changes in cirrhotic patients [19], predict therapeutic response [20, 21], and evaluate microvascular invasion [22] in HCC. However, conventional PCT involves high radiation doses, underscoring the need for low-dose PCT scanning to minimize radiation exposure and facilitate more frequent follow-ups in HCC management.

The purpose of this study was to assess the value of low-dose one-stop dual-energy and perfusion computed tomography (LD-DE&PCT)-derived parameters in predicting the efficacy of TACE-LEN-ICIs in uHCC patients.

## Materials and methods

This prospective study was approved by the Institutional Review Boards of all participating centers, and written informed consent was obtained from all participants. All patient data were anonymized throughout the study. The study was conducted in accordance with the Declaration of Helsinki and complied with all applicable ethical guidelines and regulations.

### Patients

**Fig. 1:**
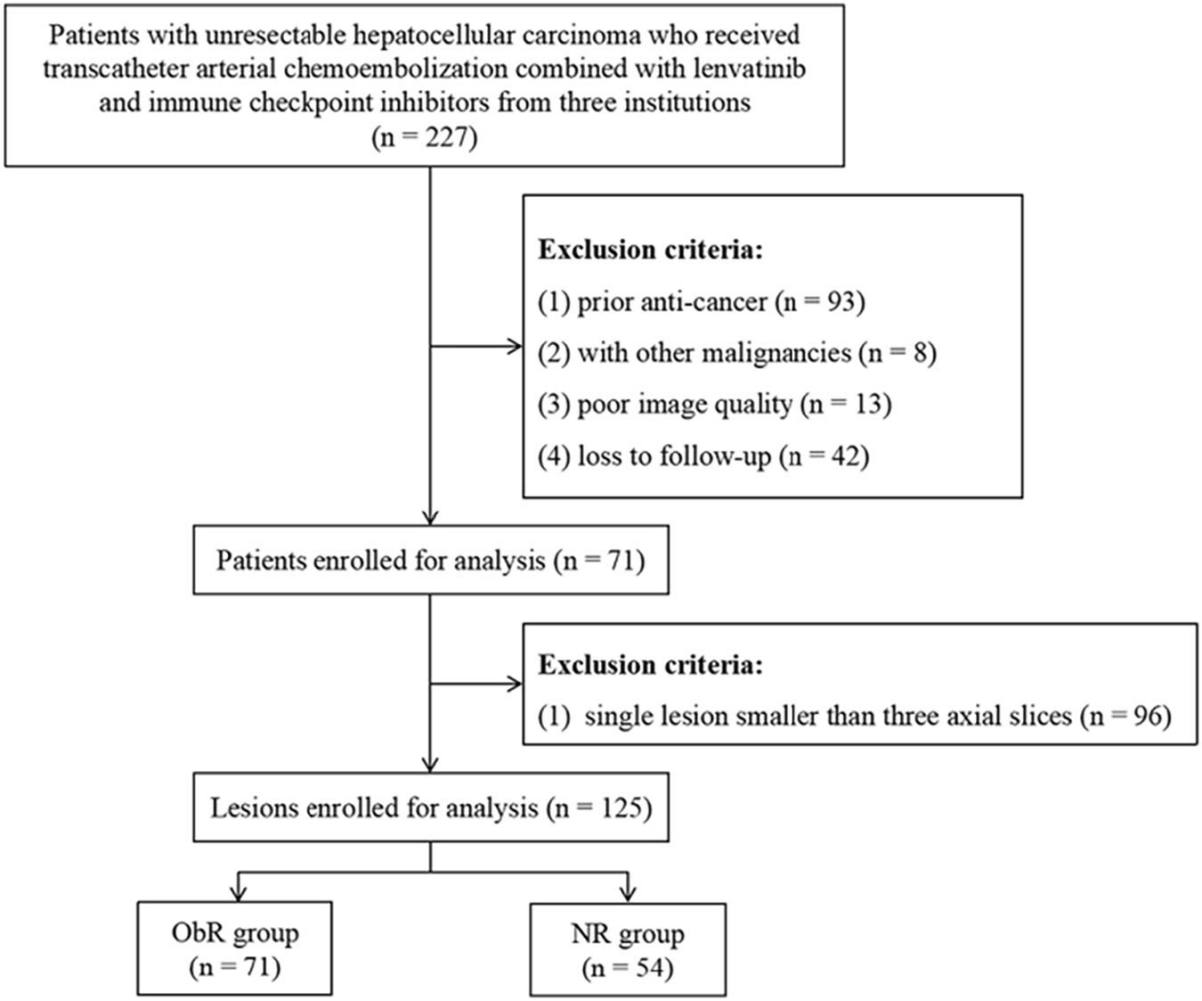
Flowchart the study. ObR, Objective response; NR, Non-response

Patients with uHCC who received TACE-LEN-ICIs between January 2024 and February 2025 were included. The inclusion criteria were as follows: (1) HCC was diagnosed clinically according to the American Association for the Study of Liver Disease criteria or the European Association for the Study of the Liver criteria [23, 24]; (2) Barcelona Clinic Liver Cancer B or C HCC; (3) age ≥ 18 years; (4) Child–Pugh class A or B; (5) TACE-LEN-ICIs administered as the first-line treatment; and (6) LD-DE&PCT performed within 2 weeks before treatment. The exclusion criteria included: (1) received other antitumor therapy; (2) a combination of other malignancies; (3) poor image quality; (4) loss to follow-up; and (5) a single lesion smaller than three axial slices. Finally, 71 patients with 125 lesions were enrolled in the study (Fig. 1).

### CT protocol

LD-DE&PCT was performed using the Revolution CT scanner (GE HealthCare, Wisconsin, USA). The routine clinical CT protocol for HCC included noncontrast, arterial phase (AP), portal venous phase (PVP), and delayed-phase scans. An unenhanced liver CT was first performed with a scan volume of 160 mm along the *z*-axis covering the entire liver.

Five seconds after intravenous injection of 70 mL of a non-ionic contrast agent (Omnipaque 350, iodine concentration (IC) 350 mg/mL, GE Healthcare) at a flow rate of 5 mL/s, followed by a 30 mL saline flush at the same rate, perfusion CT was initiated. Perfusion scans were divided into two phases: the first phase (perfusion-1), comprising 11 passes over a duration of 20.5 s, and the second phase (perfusion-2), comprising 8 passes over a duration of 21.5 s. AP scan was performed 3 s after the end of perfusion-1, and PVP scan was obtained 3 s after the end of perfusion-2.

Both AP and PVP images were obtained using the Gemstone Spectral Imaging DECT mode, which employed rapid tube voltages switching between 80 and 140 kVp with a gantry rotation speed of 0.5 s/rotation. Monochromatic DECT images were combined with conventional perfusion images to generate quantitative perfusion maps. All images were acquired with a slice thickness of 5.0 mm and a consistent field of view. Image reconstruction was performed using a standard kernel and adaptive statistical iterative reconstruction-Veo at 50% strength. The start and end positions of the perfusion and DECT images were aligned, and the slice thickness was resampled to 2.5 mm. Subsequently, these images were integrated to generate the complete PCT images. The details of scanning parameters are listed in Supplementary Table S1.

### Radiologic feature analysis

When patients had multiple lesions, we selected and assessed the two largest lesions in the liver as target lesions [25]. Two radiologists with 4 and 10 years of experience in abdominal imaging independently assessed the radiologic features. Although both radiologists were aware of the HCC diagnosis, they were blinded to all clinical information and treatment response. In cases of disagreement, a third senior radiologist with over 25 years of experience in abdominal imaging made the final decision. The definitions of all radiologic features are summarized in Supplementary Table S2.

### DECT and perfusion quantitative analysis

All quantitative measurements were performed on a workstation (AW 4.7, GE HealthCare) by two radiologists (with 5 and 6 years of experience in abdominal imaging, respectively) who were blinded to clinical information and treatment response. A dynamic correction algorithm was applied to all PCT images to eliminate motion artifacts. Two circular regions of interest (ROIs) were manually positioned in the abdominal aorta and portal vein to obtain both arterial and portal venous input functions. Subsequently, the software automatically generated various perfusion maps, providing parameters such as mean transit time, time to peak, transit time to impulse residue peak, blood volume (mL/100 g), blood flow (mL/min/100 g), hepatic arterial blood flow (mL/min/100 g), and permeability surface area product (PS, mL/min/100 g).

On the largest diameter of each lesion, three identical circular ROIs, each with an area ranging from 10.0 mm^2^ to 20.0 mm^2^, were manually delineated in the solid part of the tumor on the AP image, carefully avoiding cystic degeneration, necrotic or bleeding foci, and larger vessels. These ROIs were then automatically copied on virtual monochromatic images at 40 keV and 90 keV, IC maps, Z_eff_ maps in the AP and PVP, and the above perfusion maps to measure corresponding DECT and PCT parameters. For each parameter, the average values of the three ROIs were calculated.

An additional ROI was positioned on the abdominal aorta on the same slice, avoiding vessel wall calcifications and covering at least 50% of the arterial cross-section. Then, tumor IC values were normalized by calculating the normalized iodine concentration (NIC) using the formula: NIC = IC_tumor/IC_aorta. The slope of the spectral HU curve (λ_HU_) was calculated using the formula: λ_HU_ = (CT_40 keV_-CT_90 keV_)/(90–40).

**Fig. 2:**
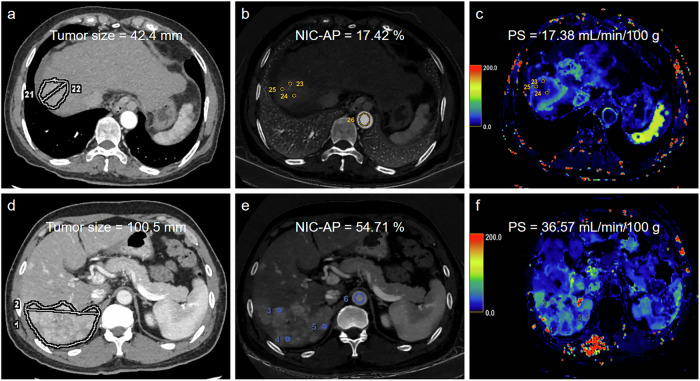
**a**–**c** A 65-year-old patient with unresectable hepatocellular carcinoma (uHCC) demonstrated an ObR to TACE-LEN-ICIs. The pretreatment AP image (**a**) showed a tumor size of 42.4 mm. The IC map (**b**) and PS map (**c**) showed normalized iodine concentration in arterial phase (NIC-AP) and PS values of 17.42% and 17.38 mL/min/100 g, respectively. **d**–**f** A 52-year-old patient with uHCC demonstrated a non-response (NR) to TACE-LEN-ICIs. The pretreatment AP image (**d**) showed a tumor size of 100.5 mm. The pretreatment IC map (**d**) and PS map (**e**) showed NIC-AP and PS values of 54.71% and 36.57 mL/min/100 g, respectively

The mean values obtained by the two observers were recorded for further analysis. Representative images are shown in Fig. 2.

### Radiation dose

The CT equipment automatically calculated the volume CT dose index‒CTDI_vol_ and dose-length product following each scan. The effective radiation dose was calculated using the dose-length product and the conversion factor of 0.015 mSv/mGy·cm, as recommended by the International Commission on Radiological Protection [26]. The formula was: Effective radiation dose = Dose-length product × 0.015 (mSv/mGy·cm).

### Treatment protocol and evaluation

Conventional TACE procedures were performed by interventional radiologists with over 10 years of experience according to TACE standardization [27].

Systemic treatments were administered 3‒14 days after the initial TACE, depending on the patients’ condition and liver function. All enrolled patients received oral lenvatinib at an initial dose of 8 mg/day for those weighing < 60 kg, or 12 mg/day for those weighing ≥ 60 kg. Intravenous infusions of ICIs (Camrelizumab 200 mg or Sintilimab 200 mg) were injected every 3 weeks. Both lenvatinib and ICIs were withheld for 3 days before each TACE session and resumed 3 days afterward, provided that no severe related adverse events occurred.

Imaging examinations and tumor marker measurements were performed at 2–3 months after the initial treatment to evaluate the treatment response using the modified response evaluation criteria in solid tumors (mRECIST) criteria [25]. Two radiologists (with 10 and 15 years of experience in abdominal imaging, respectively) assessed the treatment response of target lesions. In the event of any discrepancies between the two radiologists, a third senior radiologist with 25 years of experience in abdominal imaging was consulted to resolve any differences and reached a consensus. According to mRECIST, treatment response is categorized into four types: complete response, partial response (PR), stable disease (SD), and progressive disease (PD). ObR included complete response and PR, whereas non-response (NR) included SD and PD.

### Statistical analysis

Interobserver agreement was evaluated using the Cohen κ coefficient for qualitative variables and the intraclass correlation coefficient for quantitative parameters. Categorical variables were presented as frequencies and percentages, and comparisons between ObR and NR groups were performed using the χ^2^ test or Fisher's exact test. The normality of continuous variables was determined by the Kolmogorov–Smirnov test. Normally distributed data were expressed as mean ± standard deviation and analyzed using the independent *t*-test, while non-normally distributed data were presented as median (interquartile range) and compared using the Mann-Whitney *U*-test. Variables with statistical differences in the univariate analysis were included in a backward-stepwise multivariate logistic regression to identify independent predictors, and a predictive nomogram was constructed. Multicollinearity among variables was assessed using the variance inflation factor to ensure the independence of each variable. Receiver operating characteristic curves were depicted, and the area under the curve (AUROC), accuracy, sensitivity, and specificity were calculated to evaluate the performance of each significant parameter and nomogram.

SPSS software (version 26.0, SPSS Inc.) and R software (version 4.3.1; R Foundation for Statistical Computing) were employed for data analysis. Two-sided *p* values < 0.05 were considered statistically significant.

## Results

### General information

The characteristics of 71 uHCC patients are summarized in Table 1. Of all 71 patients with uHCC, 125 lesions were analyzed. The last follow-up time was May 15, 2025. The median follow-up duration was 78 days (range: 61‒90 days). At the follow-up examination, 71 lesions (56.8%) showed ObR, and the remaining 54 lesions (43.2%) showed NR.

**Table 1:** Demographic and clinical characteristics of patients with uHCC

Characteristic	Value (*n* = 71 patients)
Age (years)^*^	56.28 ± 9.28
Sex	
Male	63 (88.7)
Female	8 (11.3)
BMI (kg/m^2^)^*^	25.75 ± 3.87
Cirrhosis	
Present	66 (93.0)
Absent	5 (7.0)
Cause of disease	
Chronic hepatitis B	60 (84.5)
Chronic hepatitis C	3 (4.2)
Alcohol	5 (7.1)
Others	3 (4.2)
Child–Pugh class	
A	44 (62.0)
B	27 (38.0)
BCLC	
B	41 (57.7)
C	30 (42.3)
Tumor number	125
AFP (ng/mL)	
≤ 400	56 (78.9)
> 400	15 (21.1)
ALB (g/L)^*^	35.93 ± 4.60
ALT (IU/L)^†^	28.00 (20.00, 42.00)
AST (IU/L)^†^	37.00 (29.00, 48.00)
TBIL (umol/L)^†^	18.40 (13.10, 23.70)
GGT (IU/L)^†^	51.00 (31.00, 140.00)
ALP (IU/L)^*^	114.48 ± 46.80
Platelet count (×10^9^/L)^*^	128.11 ± 90.37
INR^*^	1.16 ± 0.18
PT (s)^*^	13.30 ± 1.96

Unless indicated otherwise, data are numbers of patients, with percentages in parentheses

*AFP* Alpha-fetoprotein, *ALB* Albumin, *ALP* Alkaline phosphatase, *ALT* Alanine aminotransferase, *AST* Aspartate aminotransferase, *BCLC* Barcelona Clinic Liver Cancer, *BMI* Body mass index, *GGT* Gamma-glutamyl transferase, *INR* International normalized ratio, *PT* Prothrombin time, *TBIL* Total bilirubin, *uHCC* Unresectable hepatocellular carcinoma

^*^ Data are means ± standard deviations

^†^ Data are medians, with interquartile ranges in parentheses

The conventional CT features demonstrated excellent interobserver agreement with κ-values of 0.812‒0.935 (Supplementary Table S3). The tumor size and LD-DE&PCT quantitative parameters exhibited excellent interobserver agreement with intraclass correlation coefficient values of 0.902‒0.995 (Supplementary Table S4).

### Comparison of conventional CT features and LD-DE&PCT parameters

Table 2 presents the comparison of conventional CT features between ObR and NR. Size was significantly larger in NR than in ObR (*p* = 0.018). The following two qualitative CT features were more frequently observed in NR than in ObR: corona enhancement (*p* = 0.027), and located in the peripheral zone (*p* = 0.019).

**Table 2:** Comparison of conventional imaging features between ObR and NR

Characteristic	ObR (*n* = 71)	NR (*n* = 54)	*Z/χ*^*2*^ value	*p* value
LI-RADS major imaging feature				
Nonrim APHE			0.000	1.000
Present	66 (93.0)	50 (92.6)		
Absent	5 (7.0)	4 (7.4)		
Nonperipheral washout			0.427	0.513
Present	53 (74.6)	43 (79.6)		
Absent	18 (25.4)	11 (20.4)		
Enhancing capsule			0.144	0.705
Present	30 (42.3)	21 (38.9)		
Absent	41 (57.7)	33 (61.1)		
Size (mm)^†^	33.40 (20.00, 54.80)	47.40 (23.79, 90.55)	-2.357	0.018
LI-RADS ancillary imaging feature				
Non-enhancing capsule			0.000	1.000
Present	3 (4.2)	3 (5.6)		
Absent	68 (95.8)	51 (94.4)		
Nodule-in-nodule architecture			0.098	0.754
Present	2 (2.8)	3 (5.6)		
Absent	69 (97.2)	51 (94.4)		
Mosaic architecture			3.146	0.076
Present	16 (22.5)	20 (37.0)		
Absent	55 (77.5)	34 (63.0)		
Blood products in mass			0.617	0.432
Present	4 (5.6)	6 (11.1)		
Absent	67 (94.4)	48 (88.9)		
Corona enhancement			4.881	0.027
Present	5 (7.0)	11 (20.4)		
Absent	66 (93.0)	43 (79.6)		
Non–LI-RADS imaging feature				
Tumor margin			2.875	0.090
Smooth	33 (46.5)	17 (31.5)		
Nonsmooth	38 (53.5)	37 (68.5)		
Tumor location			5.498	0.019
Peripheral zone	59 (83.1)	35 (64.8)		
Central zone	12 (16.9)	19 (35.2)		
Intratumor necrosis			2.278	0.131
Present	21 (29.6)	23 (42.6)		
Absent	50 (70.4)	31 (57.4)		
Intratumor artery			1.366	0.242
Present	53 (74.6)	45 (83.3)		
Absent	18 (25.4)	9 (16.7)		

Unless indicated otherwise, data are numbers of patients, with percentages in parentheses

*APHE* Arterial phase hyperenhancement, *LI-RADS* Liver imaging reporting and data system, *NR* Non-response, *ObR* Objective response

^†^ Data are medians, with interquartile ranges in parentheses

The DECT and PCT quantitative parameters between ObR and NR are shown in Table 3. The results showed that IC-AP, NIC-AP, Z_eff_-AP, λ_HU_-AP, and PS values were significantly higher in NR than those in ObR (all *p* < 0.05). However, no significant differences were found in other quantitative parameters.

**Table 3:** Comparison of quantitative parameters between ObR and NR

Parameter	ObR (*n* = 71)	NR (*n *= 54)	*t/Z* value	*p* value
IC-AP (mg/mL)^*^	21.22 ± 7.83	27.85 ± 11.38	-3.674	< 0.001
NIC-AP (%)^*^	17.40 ± 9.56	26.35 ± 11.98	-4.509	< 0.001
Z_eff_-AP^*^	8.79 ± 0.38	9.10 ± 0.55	-3.722	< 0.001
λ_HU_-AP^*^	2.90 ± 1.09	3.77 ± 1.55	-3.524	0.001
IC-PVP (mg/mL)^*^	20.84 ± 7.39	21.72 ± 6.97	-0.678	0.499
NIC-PVP (%)^*^	44.89 ± 12.84	48.64 ± 16.30	-1.438	0.153
Z_eff_-PVP^*^	8.79 ± 0.35	8.84 ± 0.37	-0.655	0.514
λ_HU_-PVP^*^	2.89 ± 0.98	2.99 ± 0.96	-0.581	0.562
Mean transit time (s)^*^	9.98 ± 7.38	8.45 ± 5.04	1.382	0.170
Time to peak (s)^*^	30.02 ± 5.93	28.93 ± 5.43	1.058	0.292
T_max_ (s)^*^	6.52 ± 3.18	5.87 ± 2.62	1.218	0.225
Blood volume (mL/100 g)^*^	20.04 ± 6.83	19.65 ± 8.29	0.288	0.774
Blood flow (mL/min/100 g)^†^	135.10 (73.83, 241.60)	163.60 (75.04, 229.50)	-0.257	0.797
Hepatic arterial blood flow (mL/min/100 g)^†^	68.48 (42.80, 112.30)	66.69 (51.23, 119.18)	-0.593	0.553
PS (mL/min/100 g)^*^	23.39 ± 16.08	37.37 ± 20.44	-4.281	< 0.001

*AP* Arterial phase, *CI* Confidence interval, *IC* Iodine concentration, *ICC* Intraclass correlation coefficient, *LD-DE&PCT* Low-dose one-stop dual-energy and perfusion CT, *NIC* Normalized iodine concentration, *NR* Non-response, *ObR* Objective response, *PVP* Portal venous phase, *T*_*max*_ Transit time to impulse residue peak, *Z*_*eff*_ Effective atomic number, *λ*_*HU*_ Slope of spectral HU curve

^*^ Data are means ± standard deviations

† Data are medians, with interquartile ranges in parentheses

The AUROC values for size, corona enhancement, tumor location, IC-AP, NIC-AP, Z_eff_-AP, λ_HU_-AP, and PS were 0.623, 0.567, 0.591, 0.706, 0.770, 0.711, 0.704, and 0.713, respectively. These values reflect their varying degrees of efficacy in predicting the response to TACE-LEN-ICIs in patients with uHCC. Among these parameters, the predictive performance of NIC-AP was the best, when its cutoff value was set at 21.471, the corresponding sensitivity was 0.722, the specificity was 0.803, and the accuracy was 0.768 (Table 4).

**Table 4:** Comparative efficacy of parameters in predicting response to TACE-LEN-ICIs in uHCC

Parameter	AUROC	95% CI	Sensitivity	Specificity	Accuracy	Cutoff value
Size (mm)	0.623	0.522–0.725	0.556	0.676	0.624	43.95
Corona enhancement	0.567	0.505–0.629	0.204	0.930	0.616	-
Tumor location	0.591	0.514–0.669	0.352	0.831	0.624	-
IC-AP (mg/mL)	0.706	0.611–0.802	0.630	0.746	0.696	24.575
NIC-AP (%)	0.770	0.682–0.858	0.722	0.803	0.768	21.471
Z_eff_-AP	0.711	0.616–0.806	0.593	0.803	0.712	9.044
λ_HU_-AP	0.704	0.609–0.799	0.630	0.732	0.688	3.263
PS (mL/min/100 g)	0.713	0.622–0.804	0.630	0.718	0.680	28.455

*AP* Arterial phase, *AUROC* Area under the curve, *CI* Confidence interval, *IC* Iodine concentration, *NIC* Normalized iodine concentration, *PS* Permeability surface area product, *TACE-LEN-ICIs* Transcatheter arterial chemoembolization combined with lenvatinib and immune checkpoint inhibitors, *uHCC* Unresectable hepatocellular carcinoma, *Z*_*eff*_ Effective atomic number, *λ*_*HU*_ Slope of spectral HU curve

### Construction and evaluation of the nomogram

**Fig. 3:**
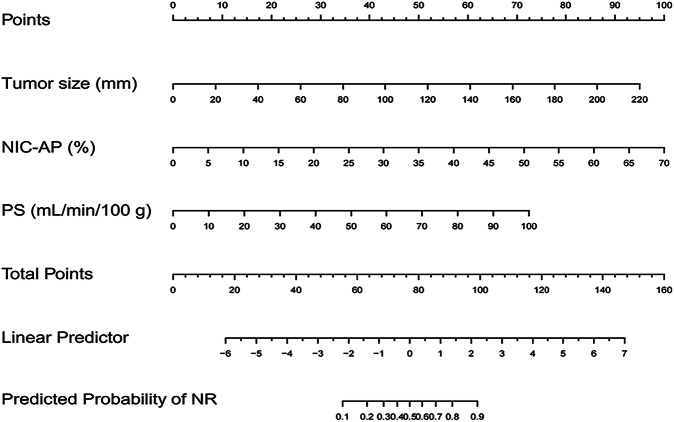
The nomogram to predict the response to TACE-LEN-ICIs in uHCC. NIC-AP, Normalized iodine concentration in arterial phase; PS, Permeability surface area product

Variance inflation factor analysis was performed to assess multicollinearity among eight variables, and the most multicollinear factor was sequentially removed until no multicollinearity existed. As a result, two variables (IC-AP and Z_eff_-AP) were excluded due to the presence of multicollinearity (variance inflation factor > 5). Consequently, size, corona enhancement, tumor location, NIC-AP, λ_HU_-AP, and PS were included in the backward stepwise logistic regression. Finally, tumor size, NIC-AP, and PS were retained as independent predictors (all *p* < 0.05), and the combined model was developed and visualized as a nomogram (Fig. 3). The nomogram was established as follows:

NR-nomogram = -7.705 + (0.043 × tumor size) + (0.143 × NIC-AP) + (0.072 × PS) (Table 5).

**Table 5:** Multivariable regression analysis of parameters

Variable	B	S.E.	Wald	OR (95% CI)	*p* value
Size (mm)	0.043	0.010	17.716	1.044 (1.023, 1.065)	< 0.001
NIC-AP (%)	0.143	0.033	18.862	1.153 (1.081, 1.230)	< 0.001
PS (mL/min/100 g)	0.072	0.017	18.054	1.075 (1.040, 1.112)	< 0.001
Constant	-7.705	1.466	27.607	-	< 0.001

After backward likelihood ratio stepwise logistic regression analysis, size, NIC-A, and PS were independent predictors. The constant term provides the baseline log odds when all predictors are zero

*AP* Arterial phase, *B* Regression coefficient, *CI* Confidence interval, *NIC* Normalized iodine concentration, *OR* Odds ratio, *PS* Permeability surface area product, *S.E*. Standard error

**Fig. 4:**
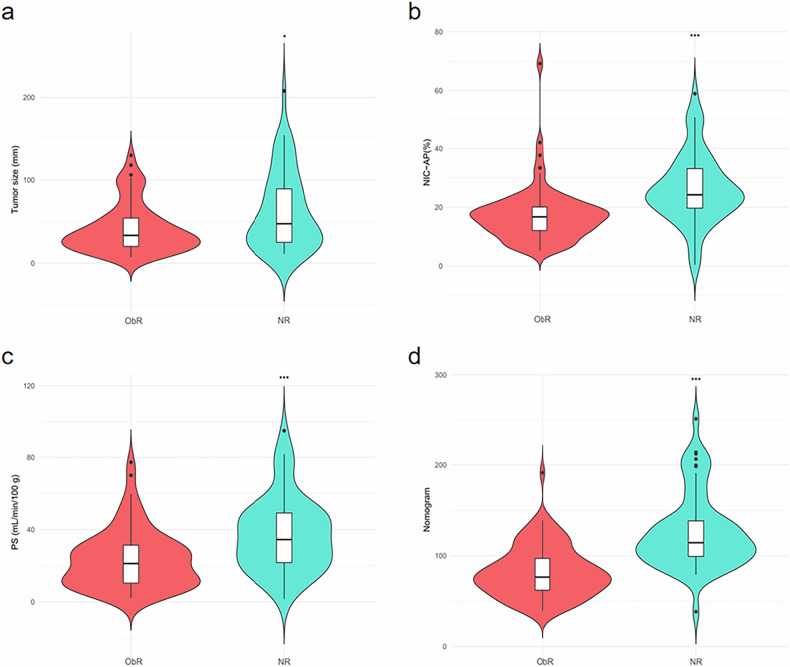
Violin plots comparing the distribution of tumor size (**a**), NIC-AP (**b**), PS (**c**), and nomogram (**d**) in the ObR and NR groups. ^*^p < 0.05; ^**^p < 0.01^;^ and ^***^p < 0.001

The distribution of the independent predictors and the combined nomogram is shown in Fig. 4.

**Fig. 5:**
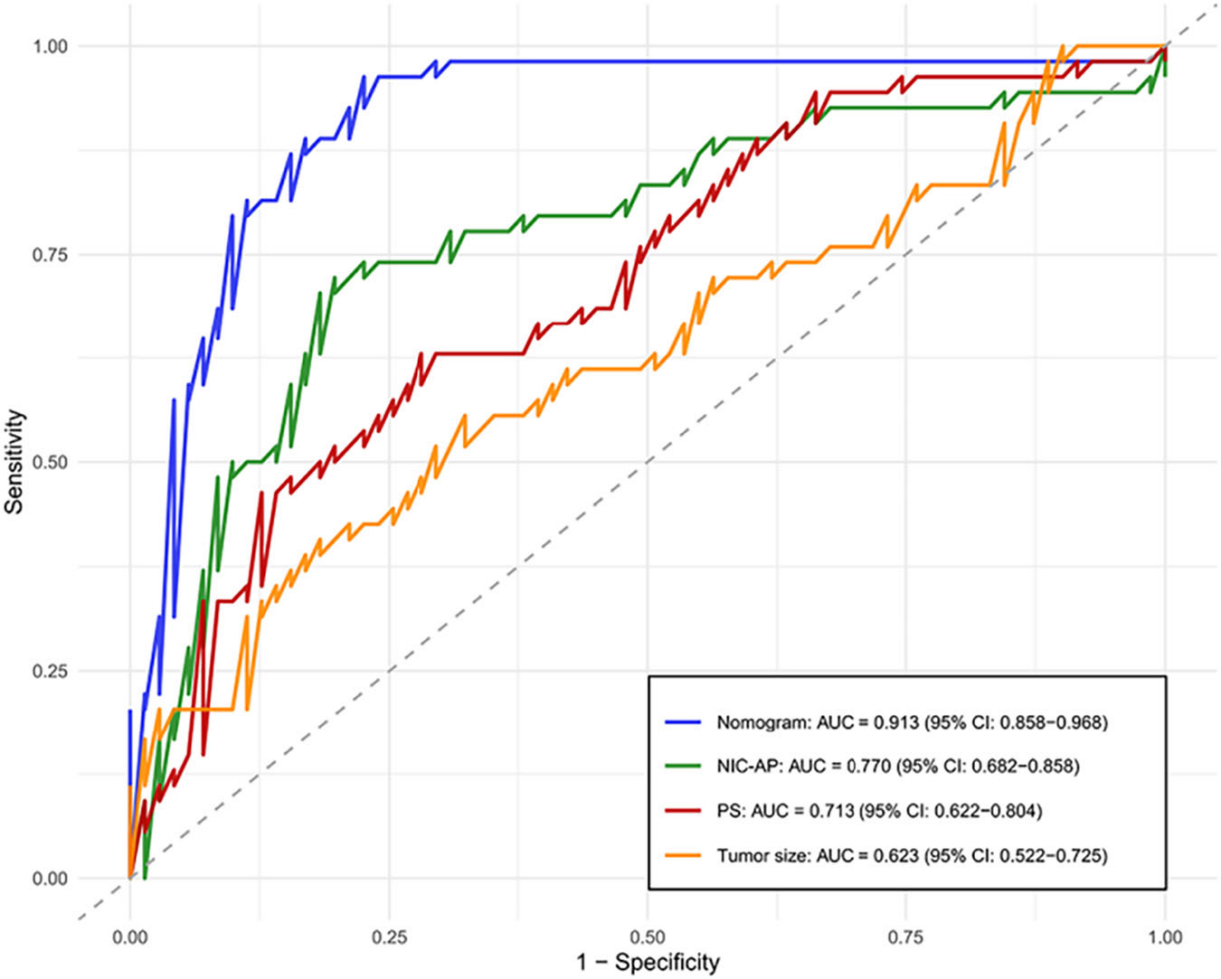
Receiver operating characteristic (ROC) curves of each independent predictor and nomogram for predicting the response to TACE-LEN-ICIs in uHCC. NIC-AP, Normalized iodine concentration in arterial phase; PS, Permeability surface area product

The receiver operating characteristic curves for these independent predictors and the combined nomogram were generated and are displayed in Fig. 5. Receiver operating characteristic analysis showed that the combined nomogram achieved the highest predictive performance, with an AUROC of 0.913 (95% confidence interval: 0.858‒0.968), significantly higher than the individual parameters (Delong test, all *p*  <  0.05). The sensitivity, specificity, and accuracy of the combined nomogram were 0.963, 0.775, and 0.856, respectively.

### Radiation dose

The average total effective radiation dose of these scans was 19.02 ± 5.39 mSv, including PCT and DECT scans, with the average total effective radiation dose of PCT being 15.55 ± 4.64 mSv.

## Discussion

Predicting the response to TACE-LEN-ICIs in uHCC patients is critical to guide treatment decisions. In this study, we developed a nomogram based on LD-DE&PCT parameters, including tumor size, NIC-A, and PS, to predict the response to TACE-LEN-ICIs in patients with uHCC. This nomogram demonstrated strong predictive performance, which could non­invasively identify candidate populations suitable for TACE-LEN-ICIs and assist clinicians in making decisions.

The synergistic antitumor mechanism of TACE-LEN-ICIs in HCC is complex. TACE induces tumor hypoxia and necrosis by embolizing the tumor-supplying arteries while directly delivering chemotherapeutic agents to the tumor. This hypoxic environment promotes tumor aggressiveness through hypoxia-inducible factor-1α-mediated pathways, whereas lenvatinib, a multi-target TKI, counteracts these effects by reducing hypoxia-inducible factor-1α levels and inhibiting angiogenesis [6, 28]. Moreover, TACE induces the release of tumor antigens and is associated with a reduction in regulatory T cells, depletion of cytotoxic T lymphocytes, and upregulation of pro-inflammatory pathways [29]. These changes help convert an immune-suppressive microenvironment into an immune-supportive one, thus enhancing the response to immunotherapy [30]. Additionally, the combination of TACE, lenvatinib, and ICIs may activate not only cellular immunity but also humoral immune responses, further augmenting the antitumor effect [31].

Several studies have emphasized the potential value of iodine content in predicting therapeutic efficacy across various cancers [32–34]. Our study identified NIC in the AP obtained using DECT as an independent predictor of response to TACE-LEN-ICIs in uHCC. NIC-AP was significantly higher in the NR group than in the ObR group, demonstrating superior predictive performance compared to other parameters, with an AUROC value of 0.770. Higher iodine content within the tumor reflects increased microvessel density and tumor angiogenesis. NIC aims to reduce variations caused by individual differences in hemodynamics and the scanning times between patients. In histopathology, disorganized neovascularization, characterized by hyperpermeable and tortuous capillaries, is a key factor contributing to increased vascular permeability. This, in turn, elevates interstitial fluid pressure, impairs perfusion, hampers the effective delivery of therapeutic agents, and ultimately reduces the therapeutic effect [35]. Moreover, higher NIC values have been linked to poor prognostic factors in HCC, such as microvascular invasion [17] and metastatic lymph nodes [36], which may indirectly suggest the aggressive behavior of the tumor. However, Ren et al reported that in nasopharyngeal carcinoma, higher NIC-A values indicated rapid blood flow delivering adequate therapeutic drugs to the tumor, thereby directly enhancing treatment efficacy [37]. These inconsistent results highlight the complex role of iodine content, which may vary depending on tumor type, vascular architecture, and treatment modality.

In our research, PS obtained using PCT was significantly elevated in the NR group, with an AUROC value of 0.713. It was also identified as an independent predictor of response to TACE-LEN-ICIs in multiparameter analysis. PS is a well-known parametric map reflecting tumor angiogenesis at varying degrees of maturation. PS refers to the rate of contrast leakage into the extracellular space; the greater the PS value is, the greater the permeability of the endothelium of newly formed microvessels in the tumor [38]. A previous study reported that PS were positively correlated with VEGF expression and microvessel density. The increase in PS in the NR group suggested an increase in intratumoral pressure, indicating lower delivery of therapeutic agents to the tumor.

Clinical characteristics typically fail to show satisfactory predictive performance. Consistent with previous results [39, 40], our study also suggested that tumor size was an independent predictor associated with treatment response, yielding a low AUROC value. To ensure the robustness of the model, we excluded multicollinearity between parameters by calculating the variance inflation factor, thus avoiding potential confounding factors. Consequently, IC-AP and Z_eff_-AP obtained through DECT were excluded from the backward stepwise logistic regression. After multivariate logistic regression analysis, the nomogram, consisting of tumor size, NIC-A, and PS, demonstrated strong performance in predicting the response to TACE-LEN-ICIs in uHCC. The nomogram achieved an area under the curve of 0.913 (95% confidence interval: 0.858‒0.968), sensitivity of 0.963, specificity of 0.775, and accuracy of 0.856. For patients who are initially diagnosed with uHCC and have not received treatment, TACE-LEN-ICIs can be considered if the nomogram indicates an effective response. However, for patients predicted to have an unfavorable response by the nomogram, other treatment options (such as bevacizumab plus atezolizumab) [41] should be recommended. For patients who fail TACE-LEN-ICIs, we advise them to receive the internationally recommended second-line treatments, such as regorafenib and apatinib [42].

Despite having reached technical maturity, PCT is rarely used in the clinical setting for the initial diagnosis of HCC. One important drawback of traditional PCT is the high radiation dose, as high temporal resolution and sufficient acquisition duration are required to accurately calculate perfusion parameters. In our study, we used 100 kVp and 150 mAs to reduce the radiation burden, and the total radiation dose of LD-DE&PCT was approximately 19.02 ± 5.39 mSv, which is lower than 32–45 mSv reported in previous liver imaging research [43, 44].

This study had some limitations. First, the relatively inadequate sample size without a validation cohort might affect the robustness of statistical analysis and limit the ability to detect significant differences. Second, clinical characteristics were not included in the model construction, as the primary focus was on lesion features on the predictive performance of the model. Incorporating additional features would require a larger sample size to prevent overfitting. Future research should address these limitations through larger cohorts, multicenter designs, and integrating clinical features to improve the reliability and generalizability of the results. Third, since China has a high incidence of hepatitis, the etiology of HCC patients included in this study was mainly viral hepatitis, whereas HCC in Western countries is predominantly alcohol-related. This distributional discrepancy in etiology might affect the global applicability of the model. Finally, as hepatectomy was not performed on uHCC, it was difficult to obtain histopathological results, making it difficult to correlate LD-DE&PCT parameters with histopathological grades.

In conclusion, a promising non-invasive model was successfully developed using the LD-DE&PCT technique to predict the efficacy of TACE-LEN-ICIs in uHCC patients. This model may identify candidates most likely to benefit from TACE-LEN-ICIs, thereby providing significant insights for clinical decision-making.

## Supplementary information

**Additional file 1: Supplementary Table S1** CT protocols. **Supplementary Table S2** Definition of each conventional CT feature in this study. **Supplementary Table S3** Inter-observer agreement for conventional CT characteristics. **Supplementary Table S4** Inter-observer reproducibility for tumor size and LD-DE&PCT parameters measurement.

## Data Availability

The data that support the findings of this study are available from LZ, but restrictions apply to the availability of these data, which were used under license for the current study, and so are not publicly available. Data are, however, available from the authors upon reasonable request and with permission of LZ.

## Abbreviations

AP: Arterial phase
CT: Computed tomography
DECT: Dual-energy CT
IC: Iodine concentration
ICIs: Immune checkpoint inhibitors
LD-DE&PCT: Low-dose one-stop dual-energy and perfusion CT
mRECIST: Modified response evaluation criteria in solid tumors
NIC: Normalized iodine concentration
NR: Non-response
ObR: Objective response
PCT: Perfusion computed tomography
PD: Progressive disease
PS: Permeability surface area product
PVP: Portal venous phase
ROI: Region of interest
TACE: Transcatheter arterial chemoembolization
TACE-LEN-ICIs: TACE combined with lenvatinib and ICIs
TKIs: Tyrosine kinase inhibitors
uHCC: Unresectable hepatocellular carcinoma
Z_eff_: Effective atomic number
λ_HU_: Slope of spectral HU curve
