# Supplementary material for: Dual-energy CT biomarkers for predicting the efficacy of TACE combined with lenvatinib and immune checkpoint inhibitors in unresectable HCC
Source: Eur Radiol Exp. 2026 Jan 19;10:4. doi: 10.1186/s41747-025-00669-9 (PMC12816452; doi:10.1186/s41747-025-00669-9)
Supplement: Supplementary file 1 — Additional file 1: Supplementary Table S1 CT protocols. Supplementary Table S2 Definition of each conventional CT feature in this study. Supplementary Table S3 Inter-observer agreement for conventional CT characteristics. Supplementary Table S4 Inter-observer reproducibility for tumor size and LD-DE&PCT parameters measurement. [file 41747_2025_669_MOESM1_ESM.pdf]

Quantitative imaging biomarkers for predicting the efficacy of transcatheter arterial chemoembolization combined with lenvatinib and immune checkpoint inhibitors in unresectable hepatocellular carcinoma

ELECTRONIC SUPPLEMENTARY MATERIAL

Supplementary Table S1 CT protocols

Scan phase	Perfusion-1	Arterial GSI	Perfusion-2	Portal GSI	Delay phase
Tube voltage (kVp)	100	80-140	100	80-140	120
Tube current (mAs)	150	405	150	405	405
Reconstructed section thickness (mm)	5/2.5	5/2.5	5/2.5	5/2.5	5/2.5
Pitch factor	0.992	0.992	0.992	0.992	0.992
Scan type	Axial	Helical	Axial	Helical	Helical

GSI, Gemstone Spectral Imaging.

Supplementary Table S2 Definition of each conventional CT feature in this study

Conventional CT feature	Definition
LI-RADS major imaging feature	
Nonrim arterial phase hyperenhancement	Nonrim-like enhancement in arterial phase unequivocally greater in whole or in part than liver. Enhancing part must be higher in attenuation or intensity than liver in arterial phase
Nonperipheral washout	Nonperipheral visually assessed temporal reduction in enhancement in whole or in part relative to composite liver tissue from earlier to later phase resulting in hypoenhancement in the portal venous phase
Enhancing capsule	Smooth, uniform, sharp border around most or all of an observation, unequivocally thicker or more conspicuous than fibrotic tissue around background nodules, and visible as enhancing rim in portal venous phase or delayed phase
Size	Largest outer-edge-to-outer-edge dimension of an observation
LI-RADS ancillary imaging feature	
Non-enhancing capsule	Capsule appearance not visible as an enhancing rim
Nodule-in-nodule architecture	Presence of smaller inner nodule within and having different imaging features than larger outer nodule
Mosaic architecture	Presence of randomly distributed internal nodules or compartments, usually with different imaging features
Blood products in mass	Intralesional or perilesional hemorrhage in the absence of biopsy, trauma or intervention
Corona enhancement	Periobservational enhancement in late arterial phase or early portal venous phase attributable to venous drainage from tumor
Non-LI-RADS imaging feature	
Tumor margin	Defined on the portal venous phase and/or delayed phase, and was categorized as i) smooth margin presenting as nodular tumors with smooth contour, and ii) non-smooth margin presenting as an irregular margin that had budding portion at the tumor periphery protruding into the liver parenchyma
Tumor location	Peripheral zone (tumors located, even in part, more than 1 cm away from the main trunk or first branch of the portal vein), and central zone (tumors located even partially within 1 cm of the main trunk or first branch of the portal vein)

Intratumor necrosis	A hypo-attenuated central area on the non-enhanced images without enhancement during the postcontrast phases
Intratumor artery	Visible blood vessel within tumor in the arterial phase

LI-RADS, Liver Imaging Reporting and Data System.

Supplementary Table S3 Inter-observer agreement for conventional CT characteristics

Characteristic	Reader 1	Reader 2	Disagreement	Kappa (95 % CI)
LI-RADS major imaging feature				
Nonrim APHE			3 (2.4)	0.812 (0.743, 0.865)
Present	118 (94.4)	115 (92.0)		
Absent	7 (5.6)	10 (8.0)		
Nonperipheral washout			4 (3.2)	0.906 (0.869, 0.933)
Present	99 (79.2)	97 (77.6)		
Absent	26 (20.8)	28 (22.4)		
Enhancing capsule			8 (6.4)	0.869 (0.819, 0.906)
Present	52 (41.6)	52 (41.6)		
Absent	73 (58.4)	73 (58.4)		
LI-RADS ancillary imaging feature				
Non-enhancing capsule			1 (0.8)	0.919 (0.887, 0.943)
Present	6 (4.8)	7 (5.6)		
Absent	119 (95.2)	118 (94.4)		
Nodule-in-nodule architecture			1 (0.8)	0.906 (0.868, 0.933)
Present	6 (4.8)	5 (4.0)		
Absent	119 (95.2)	120 (96.0)		
Mosaic architecture			5 (4.0)	0.904 (0.866, 0.932)
Present	37 (29.6)	36 (28.8)		
Absent	88 (70.4)	89 (71.2)		
Blood products in mass			2 (1.6)	0.901 (0.862, 0.929)
Present	11 (8.8)	11 (8.8)		
Absent	114 (91.2)	114 (91.2)		
Corona enhancement			4 (3.2)	0.858 (0.803, 0.898)
Present	15 (12.0)	17 (13.6)		
Absent	110 (88.0)	108 (86.4)		
Non-LI-RADS imaging feature				
Tumor margin			7 (5.6)	0.883 (0.838, 0.917)
Smooth	49 (39.2)	50 (40.0)		
Nonsmooth	76 (60.8)	75 (60.0)		
Tumor location			3 (2.4)	0.935 (0.909, 0.954)
Peripheral zone	93 (74.4)	96 (76.8)		
Central zone	32 (25.6)	29 (23.2)		
Intratumor necrosis			6 (4.8)	0.896 (0.855, 0.925)

Present	43 (34.4)	45 (36.0)		
Absent	82 (65.6)	80 (64.0)		
Intratumor artery			5 (4.0)	0.884 (0.839, 0.917)
Present	99 (79.2)	96 (76.8)		
Absent	26 (20.8)	29 (23.2)		

Note: numbers in parentheses are percentage.

APHE, arterial phase hyperenhancement; CI, confidence interval; LI-RADS, Liver Imaging Reporting and Data System.

Supplementary Table S4 Inter-observer reproducibility for tumor size and LD-DE&PCT parameters measurement

Parameter	Inter-observer reproducibility
	ICC (95% CI)
Tumor size (mm)	0.995 (0.993, 0.996)
IC-AP (mg/mL)	0.938 (0.913, 0.956)
NIC-AP (%)	0.961 (0.944, 0.972)
Z_{eff}-AP	0.902 (0.863, 0.930)
λ_{HU}-AP	0.974 (0.963, 0.982)
IC-PVP (mg/mL)	0.965 (0.951, 0.976)
NIC-PVP (%)	0.952 (0.932, 0.966)
Z_{eff}-PVP	0.941 (0.917, 0.958)
λ_{HU}-PVP	0.978 (0.969, 0.984)
Mean transit time (s)	0.981 (0.972, 0.986)
Time to peak (s)	0.947 (0.926, 0.963)
T_{max} (s)	0.958 (0.940, 0.970)
Blood volume (mL/100 g)	0.970 (0.957, 0.979)
Blood flow (mL/min/100 g)	0.969 (0.955, 0.978)
Hepatic arterial blood flow (mL/min/100 g)	0.959 (0.943, 0.971)
Permeability surface area product (mL/min/100 g)	0.967 (0.953, 0.977)

AP, arterial phase; CI, confidence interval; IC, iodine concentration; ICC, Intraclass correlation coefficient; LD-DE&PCT, low-dose one-stop dual-energy and perfusion CT; NIC, normalized iodine concentration; PVP, portal venous phase; T_{max}, transit time to impulse residue peak; Z_{eff} effective atomic number, λ_{HU}, slope of spectral HU curve.
